# Effects of Hybridization and Evolutionary Constraints on Secondary Metabolites: The Genetic Architecture of Phenylpropanoids in European *Populus* Species

**DOI:** 10.1371/journal.pone.0128200

**Published:** 2015-05-26

**Authors:** Celine Caseys, Christoph Stritt, Gaetan Glauser, Thierry Blanchard, Christian Lexer

**Affiliations:** 1 Unit of Ecology and Evolution, Department of Biology, University of Fribourg, Fribourg, Switzerland; 2 Department of Botany and Biodiversity Research Centre, University of British Columbia, Vancouver, Canada; 3 Neuchâtel Platform of Analytical Chemistry, Faculty of science, University of Neuchâtel, Neuchâtel, Switzerland; 4 Department of Botany and Biodiversity Research, University of Vienna, Vienna, Austria; Nanjing Forestry University, CHINA

## Abstract

The mechanisms responsible for the origin, maintenance and evolution of plant secondary metabolite diversity remain largely unknown. Decades of phenotypic studies suggest hybridization as a key player in generating chemical diversity in plants. Knowledge of the genetic architecture and selective constraints of phytochemical traits is key to understanding the effects of hybridization on plant chemical diversity and ecological interactions. Using the European *Populus* species *P*. *alba* (White poplar) and *P*. *tremula* (European aspen) and their hybrids as a model, we examined levels of inter- and intraspecific variation, heritabilities, phenotypic correlations, and the genetic architecture of 38 compounds of the phenylpropanoid pathway measured by liquid chromatography and mass spectrometry (UHPLC-MS). We detected 41 quantitative trait loci (QTL) for chlorogenic acids, salicinoids and flavonoids by genetic mapping in natural hybrid crosses. We show that these three branches of the phenylpropanoid pathway exhibit different geographic patterns of variation, heritabilities, and genetic architectures, and that they are affected differently by hybridization and evolutionary constraints. Flavonoid abundances present high species specificity, clear geographic structure, and strong genetic determination, contrary to salicinoids and chlorogenic acids. Salicinoids, which represent important defence compounds in Salicaceae, exhibited pronounced genetic correlations on the QTL map. Our results suggest that interspecific phytochemical differentiation is concentrated in downstream sections of the phenylpropanoid pathway. In particular, our data point to glycosyltransferase enzymes as likely targets of rapid evolution and interspecific differentiation in the ‘model forest tree’ *Populus*.

## Introduction

Plant secondary metabolites—chemical compounds not directly involved in plant growth, development, and reproduction—have a variety of roles, ranging from pigmentation to protection from ultraviolet (UV) radiation to defence against insect herbivores [1–4]. These key traits modulate many different types of ecological interactions and comprise a stunning diversity of active chemical compounds. The biochemical pathway targeted in the present study, the phenylpropanoid pathway, is estimated to represent twenty percent of all fixed carbon available in plant terrestrial ecosystems [5] and includes flavonoid, tannin, anthocyanin and lignin compounds.

The evolution of plant secondary metabolite diversity represents a topic of considerable interest in evolutionary biology and plant science [6–8]. In the current eco-evolutionary view, the origin and maintenance of plant chemical diversity is typically explained by adaptive evolution (i.e. natural selection driven by insect herbivores) and co-evolution (i.e. arms-races or frequency-dependent processes) between plant populations and interacting herbivores and pathogens [8–10]. However, changes in plant secondary metabolites due to hybridization can also affect specific biotic [11–13] and abiotic [1, 3] interactions and can have broader community effects [14]. In this study, we focus on secondary metabolite abundances in hybridizing species as functionally important suites of traits with potential links to population divergence, speciation, and species interactions.

Hybridization between species leading to taxa with ‘porous genomes’ is often observed in recently radiated groups of animals and plants [15–18]. In plants, hybridization has long been known to affect the number, diversity, and quantity of secondary metabolites [19]. Reviews of the effects of hybridization on secondary metabolism and herbivore resistance [19–21] indicate that different groups of secondary metabolites are affected by hybridization in roughly the same way, although there is still a dearth of information on chemical variation within and between different hybrid generations [21]. Typically, plant hybrids synthesize traits present in both parental species for approximately 70% of chemical traits. This high degree of additivity in hybrids, observed for most chemical traits, is complemented by less frequent phenomena such as the presence of transgressive (extreme) chemical traits, including compounds that are synthesized at higher concentrations in hybrids than in the parental species, and by the synthesis of novel compounds in hybrids. In hybridizing European *Populus* species, these proportions of traits shared between parental species and natural hybrids (comprising different hybrid generations; [22, 23]) are similar, with 50% of phenylpropanoid traits with intermediate phenotypes in hybrids, 20% of traits with parental-like phenotypes in hybrids, and 20% of traits with transgressive phenotypes [24]. While the effects of hybridization on secondary metabolites at the phenotypic level are well characterized, the genetic causes of the frequent chemical additivity and of the rarity of chemical novelty in hybrids remain largely unknown.

Plant secondary metabolism is postulated to have emerged from primary metabolism in response to environmental pressures. It is thought that gene duplication, neofunctionalization, metabolic diversification, and convergent evolution have all contributed to its emergence and expansion during the evolution of plant lineages [6, 7, 25]. Rates of metabolic enzyme evolution in plants and animals were shown to vary with position, connectivity, and metabolic flux within metabolic pathways [26, 27]. Enzymes in the upstream portions of pathways have a strong tendency to be under stronger selective constraints, to evolve more slowly, and to be more strongly pleiotropic due to their effect sizes. For example, modifications of the first enzyme of a pathway will generally have a stronger effect on the total pathway than modifications of downstream enzymes [28, 29]. In plant secondary metabolism, such differences in evolutionary constraints were found in different plants and pathways [28–31]. We apply the current knowledge and models of pathway evolution to our phenotypic data, in an attempt to further identify branch point enzymes leading to interspecific chemical differentiation within the phenylpropanoid pathway in hybridizing perennial plant species. We investigate these issues in a plant group with ongoing speciation in the face of gene flow, namely Eurasian members of the ‘model forest tree’ genus *Populus*.

We studied three natural hybrid zones and a common garden trial of *Populus alba* (White poplar) and *P*. *tremula* (European aspen), two ecologically divergent Eurasian species that form part of a species complex in section *Populus* of genus *Populus* [32]. *Populus* species (poplars, aspens, cottonwoods) represent textbook examples of forest foundation species with genetic variation in chemical defence traits that affect entire communities of herbivores and pathogens [14, 33]. Heritable variation for biotic interaction-related traits in *Populus* spp. has been found at different genetic scales, ranging from pairs of hybridizing species [14, 24, 34, 35] to local populations of the same species [36].

Using the *Populus* model, we ask three questions of current interest to the evolution of the plant secondary metabolism and defence: (1) Does the expression of secondary metabolites vary between species and geographic localities? (2) Do phytochemical defence traits in hybridizing species exhibit distinct genetic architectures involving major quantitative trait loci (QTL)? (3) Does evolution of interspecific chemical differentiation in hybridizing species involve chemical hubs and modification through enzymes with species-specific activity? To address these questions, we combine phenotyping of phenylpropanoids by liquid chromatography and mass spectrometry with molecular marker based quantitative genetic analysis of compound abundances. In particular, we examine patterns of variation in phenylpropanoid traits between species and across geographic localities, estimate the heritability of the same traits in natural populations and a common garden, and explore their genetic architecture by genetic mapping. Genetic architecture holds the key to understanding the genetic correlation structure among traits involved in biotic and abiotic interactions [37], an approach we complement by phenotypic correlation networks.

## Materials and Methods

### Plant materials and sampling design

*Populus alba* (White poplar) and *P*. *tremula* (European aspen) share broad and overlapping distribution ranges across Eurasia. The two species exhibit ecological divergence at multiple spatial scales, ranging from very large scales (*P*. *alba* reaching further south into Africa than its congener, *P*. *tremula* further north across the arctic circle) to small scales (*P*. *alba* found as pioneer in lowland flood plain forests, *P*. *tremula* primarily in upland forests) [38]. In geographic areas of overlap, *P*. *alba* and *P*. *tremula* form extensive homoploid (2n = 19) hybrid zones [22, 39]. Hybrids (a.k.a. *P*. *x canescens*) appear to be genomic mosaics with balanced genetic contributions from each parental species [22, 23], resulting in phenotypic mosaics of morphological and phytochemical traits [24, 40].

Two interconnected experiments formed the basis for this study: pheno- and genotyping of natural hybrid zones of the two species informed us on phytochemotypes in adult trees in different geographically separated populations that likely experienced spatially varying selection [41, 42], whereas a common garden trial facilitated comparison of phytochemotypes in a common and controlled environment. Sampling of natural populations comprised a total of 260 individuals (163 hybrids, 51 *P*. *alba*-like and 46 *P*. *tremula*-like trees; S1 Table) from three natural hybrid zones, referred to as (1) Ticino river (Italy), (2) Danube river (Austria), and (3) Tisza river (Hungary) valley hybrid zones [22]. Sampling permits for the Danube river (Austria) population were issued by the park authorities of the Donau-Auen National Park. No specific permits were required for the remaining locations. This study did not involve endangered or protected species. These central European hybrid zones represent differences in longitude ranging from 8.98 to 22.26^O^E from North-western Italy to eastern Hungary, but do not present large variation in latitude (between 45.28 and 48.32^O^N). The common garden trial was composed of 133 seedlings (S1 Table) from 15 open pollinated families with 5 to 17 individuals per family, planted in an unbalanced block design with randomization within blocks. The trial was established in 2011 in Fribourg, Switzerland, in typical *P*. *tremula* habitat from seeds collected from individual female trees in the Ticino river hybrid zone. The trial forms part of a larger, reciprocal common garden design involving multiple localities, to be genotyped and fully analysed at a later time. The common garden seedlings were characterized with microsatellites (S1 Materials; S2 Table) by estimating admixture proportions Q [23] and the correlation of paternity (Cp) for outcrossing species [43].

### Identification of phenylpropanoids and measurement of inter- and intra-specific geographic variation

Phenylpropanoids were previously shown to play an important role as phytochemical defence traits in *Populus* [3]. Metabolomic analysis of individual samples from natural populations [24] focussed on phenylpropanoids as biologically relevant compounds. In short, secondary metabolites were extracted from ground tissue of three silica-dried leaves per tree. 60% methanol extracts were used for both condensed tannin quantification and metabolite fingerprinting by Ultra-High-Pressure-Liquid-Chromatography Quadrupole-Time-Of-Flight Mass-Spectrometry (UHPLC-QTOF-MS) (S1 Materials). Thirty-eight compounds of the phenylpropanoid pathway were identified or tentatively identified by high-resolution mass spectrometry data and chemical standards (S3 Table). The targeted secondary metabolites were quantified in a relative manner via the normalized peak areas of chromatograms. Condensed tannins were quantified by the butanol-HCl method [27].

Inter- and intra-specific components of variation in phytochemical traits were explored by Principal Component Analysis (PCA; duality diagram function, ade4 R package plotted with ellipse function of car R package). In natural hybrid zones, raw mass spectrum data of all assayed chemical compounds (including >2800 datapoints for each individual) were compared to the selected 38 phenylpropanoids to verify correspondence of our targeted dataset with the phytochemical complexity of the total leaf extracts. In addition, PCA was used to detect interspecific and intra-specific (geographic) differentiation in each of three different subgroups of the phenylpropanoid pathway: chlorogenic acids, salicinoids, and flavonoids. The repeatability of chemical patterns in hybrid zones and the common garden was checked by comparing the positions of known pure parental and hybrid plants in principal component space. The phenotypic interspecific differentiation (S3 Table) in natural hybrid zones and the common garden was assessed with the Tukey “honest significant difference” method in R. Level of significance was fixed at adjusted P-values <0.05.

### Estimating the genetic component of phytochemical traits in natural populations and a common garden trial

The genetic component of phenotypic variation in the abundances of targeted phenylpropanoid metabolites was estimated by two different methods: (1) Mantel correlations between pairwise genomic similarities (derived from genomic admixture proportions Q, equivalent to hybrid indices) and phenotypic similarities in natural populations in the absence of pedigree information [24], based on the marker-inferred relatedness approach [44]; (2) formal estimation of heritability by half-sib analysis [45] in the common garden trial. Heritabilities (h^2^) in the common garden half-sib design were calculated from among- and within-families variance estimates [Var(s) and Var(e)] calculated with Restricted Maximum Likelihood (REML) in SPSS Statistics 20. This approach accounts for the unbalanced design resulting from variable numbers of individuals available for each family [45]. As microsatellite data indicated the presence of 31% full-siblings across the common garden families (correlation of paternity) [43], the original half-sib analysis equation was modified by multiplying the intraclass correlation by 3.

$$h^{2}=3\frac{Var(s)}{Var(s)+Var(e)}$$

### Genetic mapping of phytochemical traits in natural hybrid zones

Natural hybrid zones are considered as useful tools to study the genetic basis of trait differences between diverging populations and species [46, 47]. Therefore, we studied three natural hybrid zones [22] to assess the genetic architecture of phytochemical traits with known or suspected roles in plant defence [1, 2, 4, 35] by *in situ* genetic mapping (‘admixture mapping’). The mapping panel was identified based on the genetic admixture proportions Q (below) among >800 trees genotyped in our laboratory. The genotypic composition of the mapping panel was estimated with 77 DNA microsatellite markers from all 19 chromosomes of *Populus* [23]. Although much larger marker numbers can be obtained in these hybridizing species using genotyping-by-sequencing approaches [48], we chose to use microsatellite markers in the present study, because genetic data for them were fully available for all studied populations. Genetic data sets of this size (>40 000 data points for mapped markers from all chromosomes) are still relatively rare in studies of natural hybrid zones, and are sufficient for coarse-scale genetic mapping in recently admixed populations [49, 50].

The admixture mapping approach applied in this study is based on linear regression analysis and model selection [50]. In short, locus specific ancestries (LSA’s) were estimated for all microsatellite loci [23]. Standardized residuals from ANOVA of phenotypic data with locality as factor were calculated to account for variation among hybrid zones. Single-locus associations between phenotypes and LSA’s of each marker locus were then modelled by two generalized linear models, an additive and a dominant model, and compared using the Akaike Information Criterion (AIC). Candidate QTL with AIC≥4 were subsequently subjected to a two-step procedure of model selection (forward selection and backward elimination) to reduce false positives [50]. Percentages of Variance Explained (PVE) were estimated from linear combinations of the candidate QTL with AIC≥4 with a likelihood-ratio based pseudo-R-squared approach (r.squaredLR function from MuMIn R package).

As an indication for potential long-term selective pressures acting on these genome regions, departures from neutrality of marker loci associated with phytochemical QTL were examined in the form of interspecific heterozygote excess (an excess of heterozygotes carrying one allele of each parental species) in natural hybrids relative to the remainder of the genome [23], following the well established ‘genomic clines’ method [51]. This approach of studying departures from neutral expectations falls within a family of population genetic methods sometimes referred to as the “spy glass” approach to studying the evolutionary process, which complements the “magnifying glass” approach of studying contemporary selection with the toolbox of evolutionary ecology [52]. Interspecific heterozygote excess was extracted from genomic clines previously fitted for the same three hybrid zones [23]. Our focus was on markers with an excess of interspecific heterozygotes in the Italian hybrid zone, i.e. the population of origin of the common garden trial, and across at least two of the studied hybrid zones (Italy, Austria, and Hungary). Fisher’s exact tests were used to check whether instances of significant heterozygosity excess were more frequent for markers representing QTL for phytochemical traits than for markers from across the genome. The rationale of this test is thus similar to other QTL-based tests for selection [53, 54], with the shared caveat that the excess or dearth of particular genotype classes at the studied loci may be affected by local patterns of linkage disequilibrium (LD) along the genome.

### Phenotypic correlations of targeted phytochemicals

Previous work focusing on flavonoids in *Populus* showed clear patterns of phenotypic correlations in full-sib hybrid families [55]. In the present study, phenotypic correlation networks from the 38 studied phytochemicals identified by UHPLC-QTOF-MS (S3 Table) were used to visualize the strength of correlations between different subgroups of phenylpropanoids, and between taxa. Correlation networks do not systematically represent compound proximity within metabolic pathways, but rather represent an overview of physiological state accounting for genetic, biochemical, regulatory and environmental variations [56, 57]. Phenotypic correlations were assessed with non-parametric Spearman’s rank correlations in SPSS to account for the non-normal distribution of 34 out of 38 targeted traits. Normality of the studied compounds was assessed by the Shapiro-Wilk normality test in R. Correlation networks were generated for Spearman correlations with r>0.5 at p<0.001 using the Fruchterman-Reingold 2D algorithm (as.network function from Network and gplot function from Sna R package).

## Results

### Inter- and intra-specific variation of phytochemical traits

**Fig 1:**
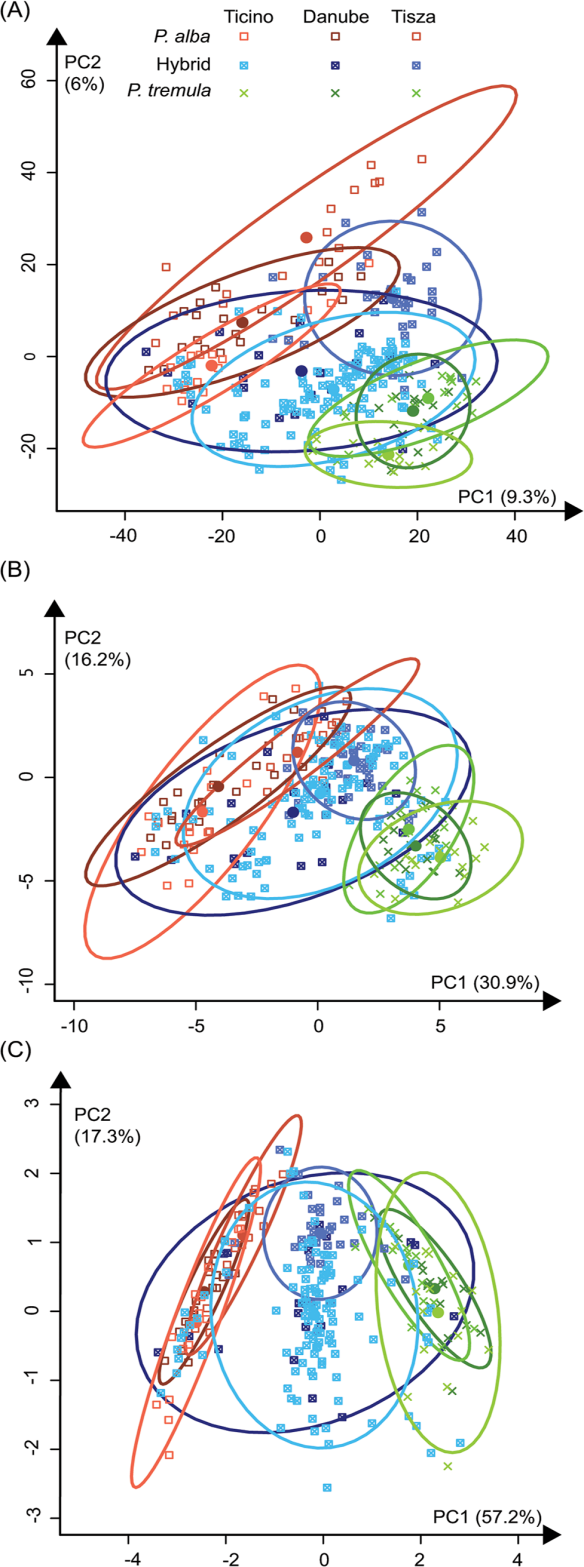
Principal component analysis of secondary metabolites in hybridizing European *Populus* species. Principal Component Analysis (PCA) of secondary metabolites identified by UHPLC-QTOF-MS in *Populus alba* (in red, square), *P*. *tremula* (in green, cross) and their hybrids, *P*. *x canescens* (in blue, crossed square). Symbols specific to each species and hues specific to hybrid zone (Ticino: Italy, Ticino river hybrid zone; Danube: Austria, Danube river hybrid zone; Tisza: Hungary, Tisza river hybrid zone) represent individuals. Ellipses represent 95% confidence intervals and coloured dots represent averages for each group. The first two principal components (PC’s) are plotted for (a) PCA of the raw mass spectrum data, i.e. all secondary metabolites detected by UHPLC-QTOF-MS, with PC1 and PC2 explaining 9.3% and 6.0% of the phenotypic variance; (b) PCA of the 38 targeted chemical traits: PC1 and PC2 explaining 30.9% and 16.2% of the phenotypic variance; (c) PCA of 18 flavonoids: PC1 and PC2 explaining 57.2% and 17.3% of the phenotypic variance, respectively.

The first two PC’s of the raw mass spectrum data (Fig 1A) accounted for 15.3% of the total variance in the three studied natural hybrid zones. The major axis of variation (PC1) resided between species, whereas PC2 captured a gradient from East (Tisza river hybrid zone, Hungary) to West (Ticino river hybrid zone, Italy; Fig 1). The first two axes from PCA of 38 phenylpropanoids (Fig 1B) accounted for 47.1% of the total variance and yielded a pattern consistent with the full metabolomic data (Fig 1A). This suggested that the targeted compounds were representative of the total variation of metabolites present in the plant extracts. The first two axes of PCA of the 18 identified flavonoids accounted for 74.5% of the total variance (Fig 1C), with clear partitioning into interspecific variation along PC1 and geographical intraspecific variation along PC2. In contrast, PCA of chlorogenic acids and salicinoids (S1 Fig) indicated that these subgroups did not present substantial inter- and intraspecific differentiation. In the common garden, the first two PC axes explained 58.9% of the total variance of the 38 phenylpropanoids (S1 Fig) and indicated the repeatability of phenotypic patterns between natural populations and the common garden trial. Overall, intraspecific chemical variation in the parental species was lower than interspecific variation (S4 Table), thus resulting in substantial differentiation of phytochemical traits between *P*. *alba* and *P*. *tremula* (Fig 1).

### Genetic component of the studied traits in natural populations and the common garden

**Fig 2:**
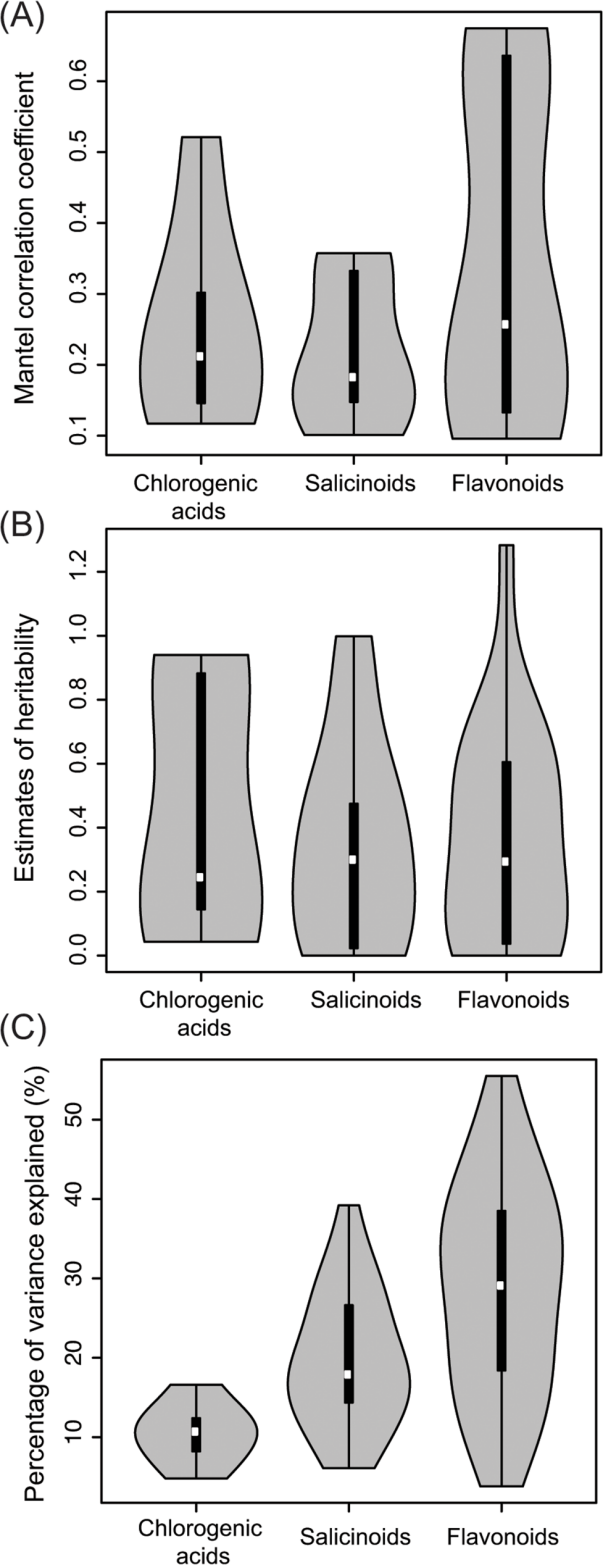
Indicators of genetic determination of phytochemical traits representing different branches of the phenylpropanoid pathway. Violin plots indicate medians (white dots), quartiles (thick black bars) and probability distributions (grey areas) of different surrogates of the degree of genetic determination or heritability in different branches of the phenylpropanoid pathway. (a) Significant Mantel correlation coefficients between phenotype and genotype in the Ticino river valley hybrid zone (Italy). (b) Estimates of heritability (h^2^) in hybrid seedlings from maternal families in the common garden. (c) Percentage of variance explained (PVE) by QTL for phytochemical traits detected by genetic mapping in natural hybrids.

Great variation in heritability across phytochemical traits was detected by both, Mantel correlations (MC) in natural populations and half-sib analysis in the common garden trial (Table 1; [24]). There was a clear tendency for greater heritability in flavonoids and chlorogenic acids compared to salicinoids. In natural populations, Mantel correlations (Fig 2A, Table 1, p<0.05) for flavonoids (mean: 0.35; range: 0.09–0.67) were larger than for chlorogenic acids (mean: 0.25; range: 0.11–0.52;) and salicinoids (mean:0.22; range: 0.1–0.35). In the common garden trial, 43% and 37% of h^2^ heritability estimates (Fig 2B; Table 1) were ≥0.50 for flavonoids (mean: 0.36; range: 0–1.28) and chlorogenic acids (mean: 0.46; range: 0.04–0.94) compared to 23% for salicinoids (mean: 0.34; range: 0–0.99). Low heritabilities (h^2^≤0.3, MC<0.2) were found for several traits of all three functional groups and were most frequent in salicinoids with low levels of substitution (salicin and salicortins) and isorhamnetin-derivatives in the flavonoid group. The h^2^ of one phytochemical trait exceeded 1, reflecting the experimental trade-off of measuring phenotypes at early life stages that matter most to tree establishment but are also most strongly affected by maternal effects [45].

**Table 1:** Thirty-eight phenylpropanoid compounds identified by UHPLC-QTOF-MS in *Populus*.

			Hybrid zone		Common garden
			All trees	Hybrid trees	Maternal families
Abbreviation	Name	Number of QTLs	Mantel Corr.	Mantel Corr.	h^2^
	**Chlorogenic acids**				
3CfQA	3-Caffeoyl quinic acid		0.164^†^	0.051	**0.926**
3CmQA	3-Coumaroyl quinic acid		0.006	0.018	**0.940**
5CfQA	5-Caffeoyl quinic acid	2	0.521^†^	0.505^†^	0.043
3fQA	3-feruloyl quinic acid	2	0.260^†^	0.248^†^	**0.839**
1CfQA	1-Caffeoyl quinic acid	1	0.316^†^	0.252^†^	0.245
5CmQA	5-Coumaroyl quinic acid	1	0.140^†^	0.083	0.184
DiCfQA	(1,5) Dicaffeoyl quinic acid	1	0.117*	0.108	0.105
	**Salicinoids**				
SC	Salicin	1	0.101*	0.011	0.017
St2	Salicortin isomer 2	1	0.041	-0.008	0.221
St3	Salicortin isomer3	1	0.046	0.034	0.139
St	Salicortin	2	0.172*	0.103	0.000
Ac-St	Acetyl-salicortin	1	0.105*	0.120	0.006
Ac-St1	Acetyl-salicortin isomer 1	4	0.148^†^	0.133*	0.475
Ac-St2	Acetyl-salicortin isomer 2	2	0.332^†^	0.196^†^	**0.998**
HCH-St	HCH-Salicortin	2	0.182^†^	0.183*	0.023
Td	Tremuloidin	4	0.348^†^	0.262^†^	0.460
Tc1	Tremulacin isomer	3	0.211^†^	0.116*	**0.564**
Tc	Tremulacin	2	0.357^†^	0.241^†^	**0.875**
HCH-Tc	HCH-tremulacin	1	0.062	0.076	0.300
Ac-Tc	Acetyl-tremulacin	1	-0.016	-0.075	0.438
	**Flavonoids**				
Cat	Catechin	2	0.236^†^	0.354^†^	0.294
Q-rut-p	Quercetin-rutinoside-pentose		0.229^†^	0.358^†^	0.000
Q-glu-p	Quercetin glucuronide-pentose	2	0.662^†^	0.661^†^	**0.739**
Q-h-p	Quercetin-hexose-pentose	2	0.639^†^	0.608^†^	**1.283**
K-rut-p	Kaempferol-rutinoside-pentose		0.096*	0.193^†^	0.000
I-rut-p	Isorhamnetin-rutinoside-pentose		0.096^†^	0.197^†^	0.000
Q-rut	Quercetin-3-O-rutinoside	1	0.553^†^	0.461^†^	0.386
Q-glu	Quercetin-3-O-glucuronide	2	0.659^†^	0.508^†^	**0.571**
Q-glo	Quercetin-3-O-glucoside	3	0.133*	0.137	0.446
K-rut	Kaempferol-3-O-rutinoside	2	0.465^†^	0.393^†^	0.265
I-rut	Isorhamnetin-3-O-rutinoside	3	0.675^†^	0.579^†^	**0.661**
Q-ara	Quercetin-3-O-arabinopyranoside	2	0.291^†^	0.296*	**0.657**
K-glu	Kaempferol-glycuronide	2	0.636^†^	0.510^†^	**0.638**
Q-rha	Quercetin-rhamnoside	3	0.038	0.031	**0.572**
I-glo	Isorhamnetin-glycoside		0.107*	0.089	0.000
I-glu	Isorhamnetin-glycuronide	2	0.184^†^	0.320^†^	0.074
I-ac-h	Isorhamnetin acetyl-hexose	1	0.084	0.092	0.098
I-rha	Isorhamnetin-rhamnoside	2	0.257^†^	0.175*	0.000
CT	Condensed tannins	3	0.113*	0.126*	0.219

Identified phytochemical traits described by their full name, abbreviation and number of QTL. Mantel correlations (Mantel corr.) between genomic similarities (derived from genomic admixture proportions Q) and phenotypic similarities [24] were used to assess the degree of genetic determination (and thus to approximate heritability) of phytochemical traits in a natural hybrid zone for all individuals including parental-like trees, and for hybrid trees only (0.01<Q<0.99). Formal estimates of heritability (h^2^) were derived from half-sib analysis using Restricted Maximum Likelihood (REML) [45] for maternal families with hybrid seedlings in the common garden. Note: *p-values<0.05, ^†^p-values<0.001, ^**‡**^ bold values indicate h^2^ values ≥ 0.500. Values >1 are most parsimoniously explained by the presence of maternal effects in 2^nd^ year seedlings.

### Genetic architecture of phytochemical traits revealed by admixture mapping in natural populations

**Fig 3:**
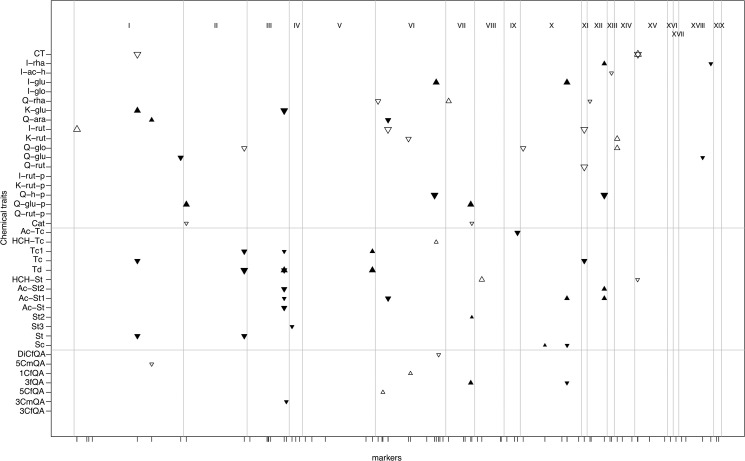
Genetic architecture of phytochemical traits inferred in natural hybrid zones of European *Populus* species. Seven chlorogenic acids, 13 salicinoids and 19 flavonoids (y axis) were tested for association with genetic markers (x axis) spread across all 19 chromosomes of *Populus* (roman numbers at the top) following an information theoretic approach [50]. For each candidate locus, additive (▼) or dominant (▲) effects are documented. Symbol sizes represent PVE’s of each candidate locus (▲<15%, ▲15–30%, ▲>30%). For details see S5 Table.

**Fig 4:**
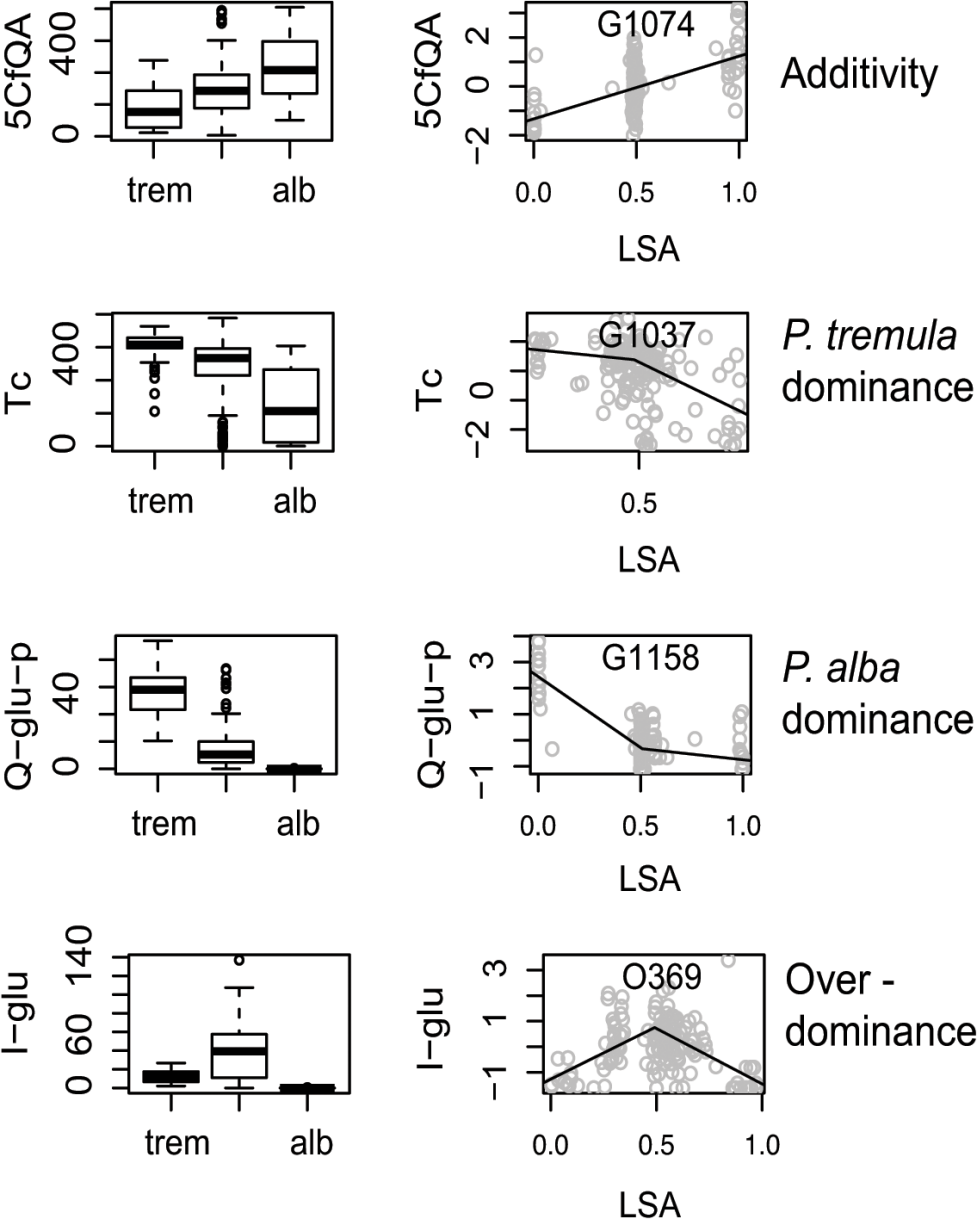
Examples of gene action in hybrids and effects on phenotype. Phenotypes for each species (left panel) and phenotype-genotype associations for four exemplary QTL (right panel; for detailed information on mapped QTL see S6 Table). Boxplots in the left panel represent medians, interquartile ranges, and outliers for phytochemical traits in *Populus tremula* (trem), *P*. *x canescens* hybrids (middle of graphs) and *P*. *alba* (alb). Scatter plots in the right panel show standardized chemical phenotypes (y-axis) plotted against Locus Specific Ancestries (LSA’s) with 0 indicating *P*. *tremula* and 1 indicating *P*. *alba* ancestry. Fitted linear regression lines indicate gene action as additive, over-dominant and dominant for alleles of one or the other parental species [50].

Single-locus tests for genotype-phenotype associations revealed one to several QTL (Table 1) for 39 metabolic traits (Fig 3; S5 Table). Out of these, 41 QTL were significant in additive and 25 in dominant generalized linear models (Fig 3). The percentages of variance explained (PVE) varied among functional groups of traits (Figs 2C and 3). The PVE’s of QTL for chlorogenic acids, salicinoids, and flavonoids exhibited means and ranges of 10.5% (4–16), 19.7% (6–34), and 28.5% (3–55), respectively (Fig 2C; S5 Table). Modes of gene action inferred from standardized residual phenotypes and LSA’s indicated over-dominance for seven, under-dominance for three, *P*. *alba*-dominance for three, *P*. *tremula*-dominance for four, and additivity for the remaining QTL (Figs 3 and 4; S5 Table). Based on results from Fisher’s exact tests, all three groups of traits exhibited significantly elevated numbers of QTL with heterozygosity excess compared to markers from across the genome in the Italian hybrid zone (S6 Table), and flavonoids did so in ≥ two hybrid zones

### Phenotypic correlations in natural populations

**Fig 5:**
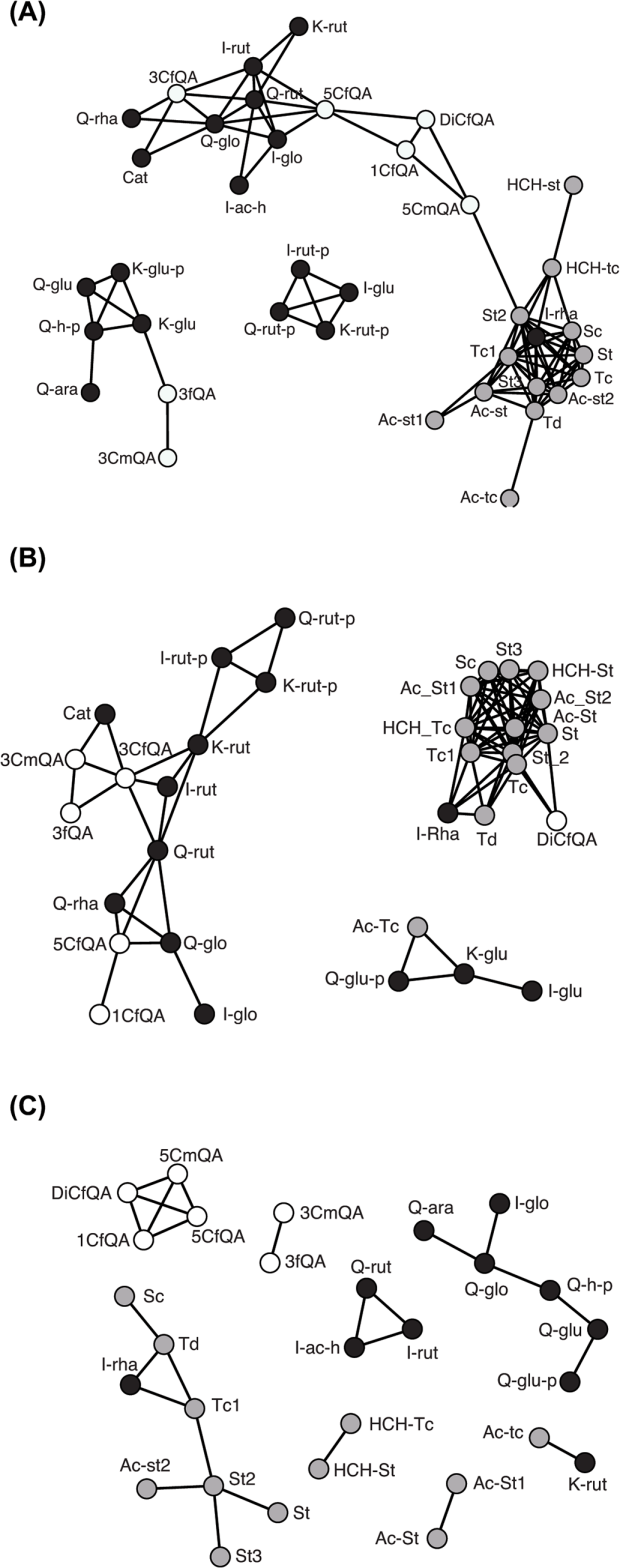
Correlation networks of phenolics in hybridizing European *Populus* species. Metabolite correlation networks of 38 phenylpropanoids (for abbreviations see Table 1) identified by UHPLC-QTOF-MS. Chlorogenic acids are coloured in white, salicinoids in grey and flavonoids in black. Correlation networks were obtained by applying Fruchterman-Reingold 2D algorithm on Spearman correlations (r>0.5, p<0.001). Metabolite correlation networks are presented for (a) admixed individuals from the Ticino river valley hybrid zone (n = 109), using thresholds of 0.05 < genomic admixture proportion Q < 0.95 to delimit admixed trees; (b) individuals of *P*. *alba* (n = 51), (c) individuals of *P*. *tremula* (n = 46) from natural hybrid zones.

Correlation networks (Fig 5) largely reflected functional relationships within each group of phytochemical traits. Trait correlations, estimated as the number of significantly (p<0.001) correlated trait pairs with r>0.5 varied between trait groups and species. In hybrids from the Ticino hybrid zone (average number of correlated pairs: 4.8; Fig 5A), salicinoids (mean: 7.0; range: 1–11) were more strongly correlated than flavonoids (mean: 3.9; range: 1–10) and chlorogenic acids (mean: 3.3; range: 1–6). Correlations patterns were similarly strong in *P*. *alba* (average number of correlated pairs: 4.4; Fig 5B) and were generally weaker in *P*. *tremula* (average number of correlated pairs: 0.9; Fig 5C). Viewed by functional groups of compounds, salicinoids comprised a tightly correlated cluster of traits, and flavonoids grouped according to sugars but not according to aglycones (core molecules of flavonoids, here Quercetin, Isorhamnetin and Kaempferol) (Fig 5). A consistent correlation between salicinoids and isorhamnetin-rhamnoside (a flavonoid) was present in all taxa.

## Discussion

Our study on the genetic architecture and phenotypic patterns of phytochemical traits in hybridizing *Populus* species differs from other recent studies on plant defence and metabolomics, which have addressed related issues primarily at the within-species level [9, 10, 36]. Our focus on phytochemical variation present in hybridizing species is of particular relevance to organismal groups in which leaky species barriers and ‘porous’ genomes are widespread [15]. The effects of hybridization on the abundances of secondary metabolites in hundreds of trees from three natural hybrid zones of European *Populus* species in our study are in line with expectations from many other plant taxa [21, 24]: hybrids present a majority of additive traits, few parental-like traits, rare transgressive traits, and little detectable chemical novelty. However, our study goes an important step beyond accounting for the phenotypic effects of hybridization by providing insights into the genetic architecture underlying these phenotypes (Figs 3 and 4). We also examined inter- and intraspecific patterns of variation for targeted phytochemical traits across geographically separated hybrid zone localities. Moreover, our phytochemical data allowed us to single out a specific gene family (glycosyltransferases) on which hybridization may have particularly strong effects.

### Inter- and intra- specific geographic variation in secondary metabolite abundances

Interspecific variation in phenylpropanoid abundances in European *Populus* species is sufficiently strong to potentially allow species identification with LC-MS techniques [24]. The first principal component axis of phenylpropanoid traits (Fig 1 and S1 Fig) is highly correlated with a hybrid index measured with molecular techniques such as microsatellites [23, 24] or second-generation sequencing based markers [58]. Still, intraspecific variation is an important factor, since plant defences and secondary metabolism are known to exhibit geographic variation [10, 59, 60]. Geographic structure in defence genes, metabolome traits and herbivore communities has recently been documented in Swedish populations of *P*. *tremula* [36]. However, no geographic structure was found for salicinoids in the same set of populations [61]. Here, we explored geographic patterns of phytochemical traits along a longitudinal transect of three natural hybrid zones of *P*. *tremula* and *P*. *alba*. We found no evidence of geographic structure for chlorogenic acids and salicinoids, but strong geographic structure for flavonoids in the studied species and their hybrids (*P*. *x canescens*). These differences in geographic structure between different subgroups of phenylpropanoids can plausibly be explained by (1) differences in levels of available genetic variation among phytochemical traits representing these subgroups of the pathway [10, 60] and/or (2) differential involvement of these subgroups in genotype x environment (GxE) interactions, i.e. local adaptation [62].

Our genetic mapping results show that flavonoids exhibit a stronger genetic component (e.g. larger PVE’s; Fig 2C) than salicinoids and chlorogenic acids. The apparent geographic pattern from East to West seen in these species and hybrids (Fig 1) suggests that phytochemical trait expression follows similar geographic patterns as previously seen for neutral genetic markers, presumably caused by patterns of postglacial recolonization [22, 41, 63].

Although the presence of genetic (= heritable) variation alone may explain geographic structure as observed for flavonoids in the present study (Fig 1C), local adaptation remains a suitable alternative hypothesis. Whereas salicinoids are frequently discussed as typical anti-herbivory compounds in Salicaceae, flavonoids also fulfil more general functions by conferring both biotic and abiotic protection, especially in the context of water stress (hydrology, precipitation) and UV radiation [1, 64]. In addition, the large flavonoid molecules known as condensed tannins are textbook examples for traits with ‘extended phenotypes’, thus suggesting the ecological importance of this subgroup of the phenylpropanoid pathways in *Populus* [65]. Reciprocal transplant experiments would allow specific tests for the potential roles of these phytochemical traits in local adaptation [62].

### Genetic architecture and phenotypic correlations of phenylpropanoids

Coarse-scale genetic mapping based on genetic markers from all 19 poplar chromosomes provided a glimpse of the genetic architecture of 38 phenolics identified and quantified by UHPLC-QTOF-MS and condensed tannins (Fig 2; S4 Table). In particular, the results are consistent with the presence of ‘major genes’ controlling species differences likely arisen by divergent natural selection or a combination of selection and drift [50, 66]. We have studied genetic variation segregating between highly divergent species, which is expected to uncover more distinct genetic architectures than genetic mapping of traits at the within-species level [67]. Our results are generalizable to the extent that genome admixture between taxa with porous genomes (Fig 4) plays a role in ecology and evolution [15, 16, 66].

**Fig 6:**
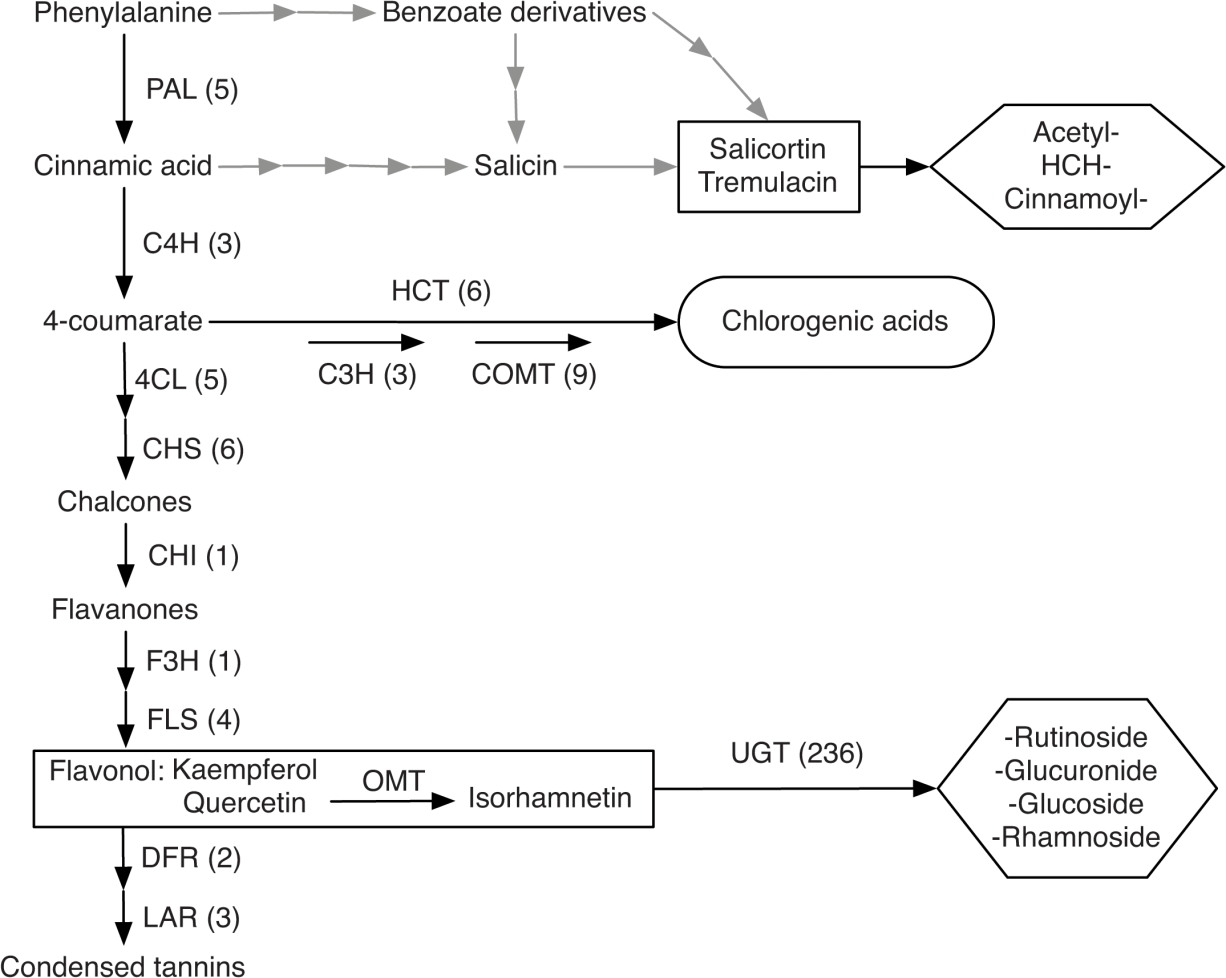
Overview of the phenylpropanoid pathway. Black arrows represent well-known branches of the phenylpropanoid pathways, their enzymes and numbers of genes in *Populus* [70, 71]. The hypothesized metabolite network for salicinoids is represented by grey arrows, following [72]. Common aglycones are represented in square boxes and moieties in hexagonal boxes. PAL, Phenylalanine ammonia lyase; C4H, cinnamate 4-hydroxylase; 4CL, 4-coumarate: CoA ligase; CHS, chalcone synthase; CHI, chalcone isomerase; F3H, flavanone 3-hydroxylase; FLS, flavonol synthase; DFR, dihydroflavonol reductase; LAR, leucoanthocyanindin reductase; HCT, hydroxycinnamoyl-CoA quinate/shikimate hydoxycinnamoyltransferase; C3H, 4-coumarate-3-hydroxylase; COMT: Caffeate *O*-methyltransferase; OMT: *O*-methyl transferase; UGT, UDP-glycosyltransferase.

As in most QTL mapping studies of wild species, PVE’s in our study must be regarded as upper bounds [68]. In addition, the sample sizes employed in this study certainly allowed us to see only the “tip of the iceberg” of the QTL distribution for each trait [67]. Future studies based on greater sample sizes and marker numbers [48] will provide a more fine-grained picture of the genetic architecture of ecologically important traits in hybridizing species, especially in more highly recombinant mapping populations [58]. Within the limits of statistical power [50] afforded by the current mapping study, our results indicate high heritability (Table 1) and a relatively simple architecture of leading QTL for many of the studied phytochemical traits (Fig 2; S5 Table). QTL effect sizes (PVE’s) were on average greater for flavonoids than for salicinoids and chlorogenic acids. This trend across functional categories of chemical traits, largely consistent with our heritability estimates (Table 1), suggests that our mapping results are robust. Also, loci for two salicinoids (salicortin and HCH-salicortin) mapped to the same approximate locations in a QTL mapping pedigree of North American *Populus* species (Scott Woolbright, pers. comm.), which further validates our results obtained by admixture mapping. In the following paragraphs, we detail our findings for each branch of the phenylpropanoid pathway (Fig 6) regarding genetic architecture, genetic and phenotypic correlations, and the potential effects of genotypes and the environment.

#### Chlorogenic acids

This group of secondary metabolites is involved in defence against herbivores, and their effect is enhanced by peroxidases [69]. Heritabilities and QTL effect sizes of these traits in our study (Fig 2) were low and there was little phenotypic variation between species (S1 Fig). Minor QTL, explaining small proportions of the phenotypic variation in each trait, were found spread across five chromosomes with three QTL positioned on chromosome VI (Fig 3). Phenotypic correlation networks suggest that chlorogenic acids are associated with intermediate compounds of the flavonoid pathway (Fig 5A and 5B), in agreement with available models of the phenylpropanoid pathway (Fig 6), whereas correlations with salicinoids were weak.

#### Salicinoids

Salicinoids are ecologically important anti-herbivory compounds in the Salicaceae family [4]. Although salicinoids are described as a defined branch of the phenylpropanoid pathway [70, 72], the exact sequence of steps and molecules and their respective intermediate forms remain largely unknown. It has been proposed that salicinoids may not be synthesized within a single linear pathway but rather within a network of interconnected reactions (Fig 6; [72]). The high phenotypic correlations found in this group (Fig 5) suggest particularly strong dynamics within the pathway, with compounds functioning as substrates of one another. As [61] proposed, the dynamics within the salicinoid pathway might depend on the availability of moieties (acetyl-, Cinnamoyl- and HCH-derivatives) with common salicinoids (salicortin and tremulacin; Fig 6) most likely stocked as intermediates with various isomer forms. In this study, we provide first evidence of the coarse-scale genetic architecture of phytochemical traits in this pathway with 15 different QTL detected, six of which control the abundances of two or more salicinoid compounds (Fig 3). The abundance of many salicinoids exhibited genetic correlations (linkage and/or pleiotropy) visible as genomic clustering on the QTL map on chromosomes II and III (Fig 3). This suggests that the entire pathway may either be under the control of few regulatory genes or elements, or that salicinoid synthesis involves few enzymes, as is the case for the terpenoid pathway [61]. Chemical links with the phenylpropanoid pathway are suggested by phenotypic correlations with the abundances of chlorogenic acids, but also with the abundance of a flavonoid, Isorhamnetin-rhamnoside (Fig 5), which is mirrored by QTL co-localization on chromosome XII (Fig 3).

#### Flavonoids

Flavonoids represent a large group of phytochemicals widely distributed in the plant kingdom [1]. In European *Populus* species, some flavonoids, especially the glucuronide and rutinoside derivatives, present high heritability and species specificity (S2 Fig, [24]). Estimates of both, flavonoid heritability and QTL effect sizes (PVE; Fig 2) were on average larger than for chlorogenic acids and salicinoids. Phenotypic correlations (Fig 5) were detectable between compounds sharing glycosylation patterns, but not between aglycones. QTL for flavonoid abundances mapped to twelve of the 19 *Populus* chromosomes (Fig 3) with up to two QTL co-localizations (Table 1), but without discernable genetic correlation patterns. Thus, the genetic architecture of flavonoids appears to be more complex, with many QTL of intermediate to large effects. Flavonoids were largely species-specific and exhibited geographic variation (see above), consistent with genetic control and GxE. Heritable variation for condensed tannin (= large flavonoid molecules) abundances has previously been shown to result in measurable ‘extended phenotypes’ of *Populus* and other tree species at the level of terrestrial, aquatic, and endophyte communities and even at the level of entire ecosystems (nutrient cycling; [14]).

#### Excess of heterozygosity associated with phytochemical QTL

A growing body of literature reports the ability of herbivore and pathogen communities to drive rapid evolution in short-lived host plant species [9, 10]. Here, we explore this issue for long-lived trees representing foundation species of terrestrial ecosystems. A previous study has shown that naturally occurring hybrid populations between *P*. *alba* and *P*. *tremula* retain unexpectedly high levels of interspecific heterozygosity (individuals with hetero-specific allele combinations) at many loci in the genome [23], despite several (95% CI = 23–43) generations of recombination between the parental species [50]. Selection for increased between-species heterozygotes was put forward as a likely explanation, although [23] were cautious to pinpoint the precise mechanisms responsible. Markers identified as QTL for salicinoid and flavonoid abundances in the present study exhibited a significant enrichment for loci with such ‘heterozygote excess’ in the Italian hybrid zone. For both functional groups of traits, Fisher’s exact tests indicated that instances of ‘heterozygote excess’ among phytochemical QTL were more numerous than expected from all marker loci typed by [23]. When the test was repeated across hybrid zone localities, a significantly increased count of ‘heterozygote excess’ QTL was still observed for flavonoids, but not for salicinoids (S5 Table). The results suggest selection as a potential driver of the genotypic composition of these trees. By extension, genetic variation associated with flavonoids appears to be more consistently selected compared to salicinoids. Future work should aim to identify the genetic mechanisms responsible (i.e. heterosis or alternative hypotheses; [21]) and the precise biotic or abiotic selective agents.

### Evolutionary constraints and chemical diversification of phenylpropanoids in *Populus*

*Populus alba* and *P*.*tremula* are hybridizing, ecologically divergent species with porous species boundaries [22, 23, 40, 50] that nevertheless remain differentiated in their secondary metabolism (Fig 1 and S1 Fig). We propose a prediction-based model that follows pathway evolution theory [26, 27] and accommodates experimental results [28–31] to explain and further identify targets of differentiation in the phenylpropanoid pathway.

Changes in evolutionary rates with pathway position and flux control have been described in plant secondary metabolites, with selective constraints decreasing along pathways [26, 30, 73]. This model of evolution of the plant metabolism suggests differences in evolutionary constraints along the phenylpropanoid pathway that match well with the phenotypic results observed here. Chlorogenic acids and salicinoids present low levels of species differentiation (S1 Fig; S4 Table). On the contrary, some flavonoids present high levels of interspecific differentiation (Fig 1 and S2 Fig; S4 Table). These results suggest high conservation of branches in upstream portions of the pathway (i.e. chlorogenic acids and salicinoids) and point to the potential for chemical differentiation downstream in the flavonol branch of the flavonoid pathway.

As described in [24] and plotted in S2 Fig, flavonoids in the two parental species are far less differentiated in aglycones (core of molecules, square boxes in Fig 6) than in moieties (functional groups or sugars linked to the aglycone; hexagonal boxes in Fig 6). Similarities in total concentrations between species (S2 Fig) in the face of high chemical diversity (Table 1) of flavonol suggests that the flavononol aglycone pathway is conserved and that interspecific differentiation occurred by further modification of aglycones. Also note that the transgressive traits identified in the hybrids present an unusual flavonol moiety (rutinoside-pentose). This suggests that genes involved in the addition of moieties to aglycones are particularly affected by interspecific differentiation and hybridization. Two possible scenarios may explain moiety variation: (1) the chemical diversity in salicinoids and flavonoids is driven by the availability of moieties (e.g. different sugars) in different species and ecotypes; (2) the chemical diversity is driven by enzymes catalysing the transfer of moieties to aglycones and by their specificity. We argue in favour of the second scenario and propose that such enzymes are disproportionately affected by interspecific differentiation and hybridization.

Enzymes transferring sugar moieties, socalled glycosyltransferases, have been characterized in many plant species. Glycosyltransferases fulfill various functions during compound biosynthesis, storage and regulation and present high levels of genetic diversity and complexity [74, 75]. The UDP glycosyltransferase (UGTs) genes in *Populus* represent a large family of 236 identified genes (Fig 6), twice the number of UGT genes observed in *Arabidopsis thaliana* [71]. Among these genes, Flavonoid-3-O-glycosyltransferase (9 genes) and flavonol 7-O-glycosyltransferase (27 genes) present important and highly diverse gene families in *Populus* [71]. Glycosyltransferases have also been shown to exhibit regulatory activity on diverse enzymes of the phenylpropanoid pathway. In potato, for example, a UGT has been shown to control the anthocyanin pathway after over-expression of dihydroflavonol reductase [76]. In *Populus*, over-expression of a UGT with low substrate specificity targeting cinnamic and benzoic acids is known to modulate the phenylpropanoid carbon flow without interfering with the expression levels of genes of the pathway [77]. Thus, it appears likely that UGTs are responsible for the inter- and intraspecific patterns of variation observed for flavonoids in our study (Figs 1 and 2).

## Conclusions

Phytochemical traits from different branches of the phenylpropanoid pathway differ greatly in proportions of intra- and interspecific variation, heritabilities, genetic architectures, and phenotypic correlations in hybridizing Eurasian *Populus* species. Flavonoids exhibit stronger differentiation between species and geographic localities, stronger G components, and larger numbers of detected QTL with intermediate to large effect sizes compared to most other studied phenylpropanoids. Inter- and intraspecific differentiation for the abundances of flavonoids can plausibly be explained by both, relaxed selective constraints and increased numbers of adaptive substitutions for sugar-transferring enzymes (glycosyltransferases), a gene family with greatly increased diversity and complexity in *Populus*. Our results demonstrate how different groups of secondary metabolites are differentially affected by evolutionary constraints and hybridization in plant taxa with porous genomes. Future work should combine the metabolomic approach taken here with proteomics, transcriptomics, and high-density genotyping of appropriate association mapping panels phenotyped in controlled environments. This combination of approaches should help towards closing current knowledge gaps regarding the origin and maintenance of functionally important phenotypic differences during plant population divergence and speciation.

## Supporting Information

S1 Fig Principal component analysis of chlorogenic acids and salicinoids in natural populations and a common garden trial. Principal Component Analysis (PCA) of secondary metabolites identified by UHPLC-QTOF-MS in *Populus alba* (in red), *P*. *tremula* (in green) and their hybrids, *P*. *x canescens* (in blue). Ellipses represent the 95% confidence intervals and colored dots represent averages for each group. For (A) and (B), symbols specific to each species and hybrid zone (Ticino: Italy, Ticino river hybrid zone; Danube: Austria, Danube river hybrid zone; Tisza: Hungary, Tisza river hybrid zone) represent individual trees. The first two principal components (PC’s) are plotted for (A) PCA of seven chlorogenic acids explaining 71.6% of the phenotypic variation; (B) PCA of the 13 salicinoids explaining 85.1% of the phenotypic variance within this subgroup; (C) PCA of all 38 phenylpropanoid in 133 individuals from the common garden explaining 58.9% of the phenotypic variance. For PCA for flavonoids see main paper.
(PDF)

S2 Fig Interspecific variation for total flavonoids, quercetin and kaempferol aglycones, rutinoside and glucuronide moieties. Normalized peak areas used as measures of relative abundances of compounds were summed within groups of aglycones and moieties for *P*. *alba*, *P*. *tremula* and their hybrids from three natural hybrid zones. For compound diversity see S3 Table.
(PDF)

S1 Materials A) High-throughput quantification of phenylpropanoids in natural populations. B) Molecular genetic analysis of common garden seedlings.
(PDF)

S1 Table Number of individuals studied in three natural hybrid zones. Hybrid individuals and plants from each parental species (*P*. *alba* and *P*. *tremula*) were characterized with 77 microsatellite DNA markers in a previous study [23]. The common garden plants were genotyped with 16 microsatellites for the present study (see text and S2 Table for details). Assignment to each taxon was based on Bayesian genomic admixture proportions Q as described in main text.
(PDF)

S2 Table Molecular marker used to characterize common garden seedlings. Information for the 16 microsatellite marker loci used to identify parental species and hybrids and to estimate the correlation of paternity (Cp) in the common garden. These 16 microsatellites are a subset of the genome-wide marker panel used for admixture mapping in natural hybrid zones and are fully described [23]. Localization on chromosomes, allele frequency differential (delta) between the parental reference populations of the Italian hybrid zone [23], number of alleles (N_A_) and gene diversity (He) in the common garden trial are indicated.
(PDF)

S3 Table Details of thirty-eight phenylpropanoids identified [24] by Ultra-High Pressure Liquid Chromatography coupled with Quadrupole-Time Of Flight mass spectrometry (UHPLC-QTOF-MS) in *P*. *alba*, *P*. *tremula* and their hybrids. (XLSX)

S4 Table Intra- and inter-specific variation in 38 phytochemical traits identified by UHPLC-QTOF-MS and condensed tannins. Shown are means ± standard deviations of traits quantified for three natural hybrid zones (Italy, Austria, Hungary) and a common garden trial established from seeds from the Italian hybrid zone. Numbers of individuals (n) for each species in each hybrid zone are indicated.
(XLSX)

S5 Table Genomic architecture of phytochemical traits inferred from admixture mapping in natural hybrid zones. (XLSX)

S6 Table Excess of interspecific heterozygosity linked with phytochemical QTL. Contingency tables presenting counts of the presence (yes) or absence (no) of an excess of interspecific heterozygote [23], for all 67 codominant genetic markers (S5 Table) studied in natural hybrid zones of *P*. *alba* and *P*. *tremula*, for markers representing putative QTL for salicinoids and flavonoids, and for all 34 markers representing putative phytochemical QTL in the present study.
(PDF)
