# Supplementary material for: Effects of Hybridization and Evolutionary Constraints on Secondary Metabolites: The Genetic Architecture of Phenylpropanoids in European Populus Species
Source: PLoS One. 2015 May 26;10(5):e0128200. doi: 10.1371/journal.pone.0128200 (PMC4444209; doi:10.1371/journal.pone.0128200)
Supplement: S1 Fig — Principal Component Analysis (PCA) of secondary metabolites identified by UHPLC-QTOF-MS in Populus alba (in red), P. tremula (in green) and their hybrids, P. x canescens (in blue). Ellipses represent the 95% confidence intervals and colored dots represent averages for each group. For (A) and (B), symbols specific to each species and hybrid zone (Ticino: Italy, Ticino river hybrid zone; Danube: Austria, Danube river hybrid zone; Tisza: Hungary, Tisza river hybrid zone) represent individual trees. The first two principal components (PC’s) are plotted for (A) PCA of seven chlorogenic acids explaining 71.6% of the phenotypic variation; (B) PCA of the 13 salicinoids explaining 85.1% of the phenotypic variance within this subgroup; (C) PCA of all 38 phenylpropanoid in 133 individuals from the common garden explaining 58.9% of the phenotypic variance. For PCA for flavonoids see main paper. (PDF) [file pone.0128200.s001.pdf]

S1 Figure. Principal component analysis of chlorogenic acids and salicinoids in natural populations and individuals of a common garden trial.

Principal Component Analysis (PCA) of secondary metabolites identified by UHPLC-QTOF-MS in *Populus alba* (in red), *P. tremula* (in green) and their hybrids, *P. x canescens* (in blue). Ellipses represent the 95% confidence intervals and colored dots represent averages for each group. For (A) and (B), symbols specific to each species and hybrid zone (Ticino: Italy, Ticino river hybrid zone; Danube: Austria, Danube river hybrid zone; Tisza: Hungary, Tisza river hybrid zone) represent individual trees. The first two principal components (PC's) are plotted for (A) PCA of seven chlorogenic acids explaining 71.6% of the phenotypic variation; (B) PCA of the 13 salicinoids explaining 85.1% of the phenotypic variance within this subgroup; (C) PCA of all 38 phenylpropanoid in 133 individuals from the common garden explaining 58.9% of the phenotypic variance. For PCA for flavonoids see main paper.

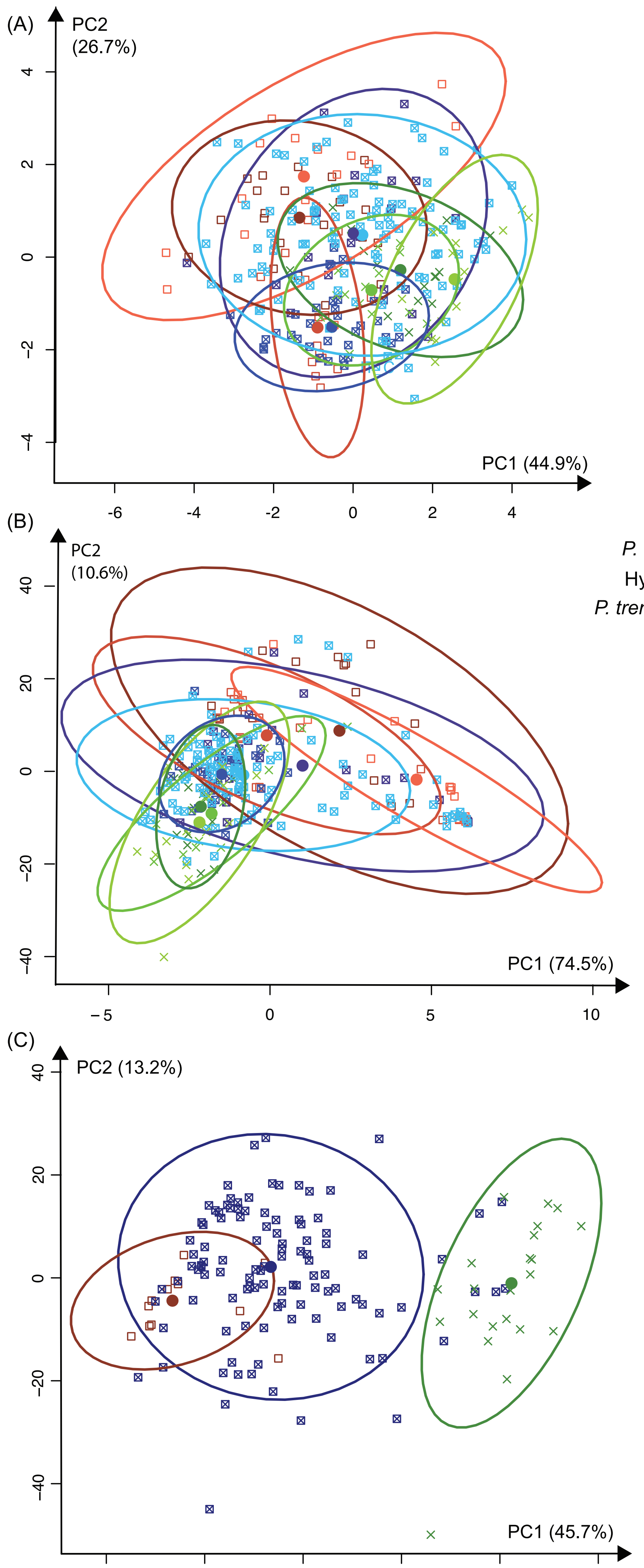
(A)
PC2 (26.7%)
PC1 (44.9%)
(B)
PC2 (10.6%)
PC1 (74.5%)
Ticino
Danube
Tisza
P. alba
Hybrid
P. tremula
(C)
PC2 (13.2%)
PC1 (45.7%)
