# Supplementary material for: Effects of Hybridization and Evolutionary Constraints on Secondary Metabolites: The Genetic Architecture of Phenylpropanoids in European Populus Species
Source: PLoS One. 2015 May 26;10(5):e0128200. doi: 10.1371/journal.pone.0128200 (PMC4444209; doi:10.1371/journal.pone.0128200)

S2 Figure. Interspecific variation for total flavonoids, quercetin and kaempferol aglycones, rutinoside and glucuronide moieties.

Normalized peak areas used as measures of relative abundances of compounds were summed within groups of aglycones and moieties for *P. alba*, *P. tremula* and their hybrids from three natural hybrid zones. For compound diversity see S3 Table.

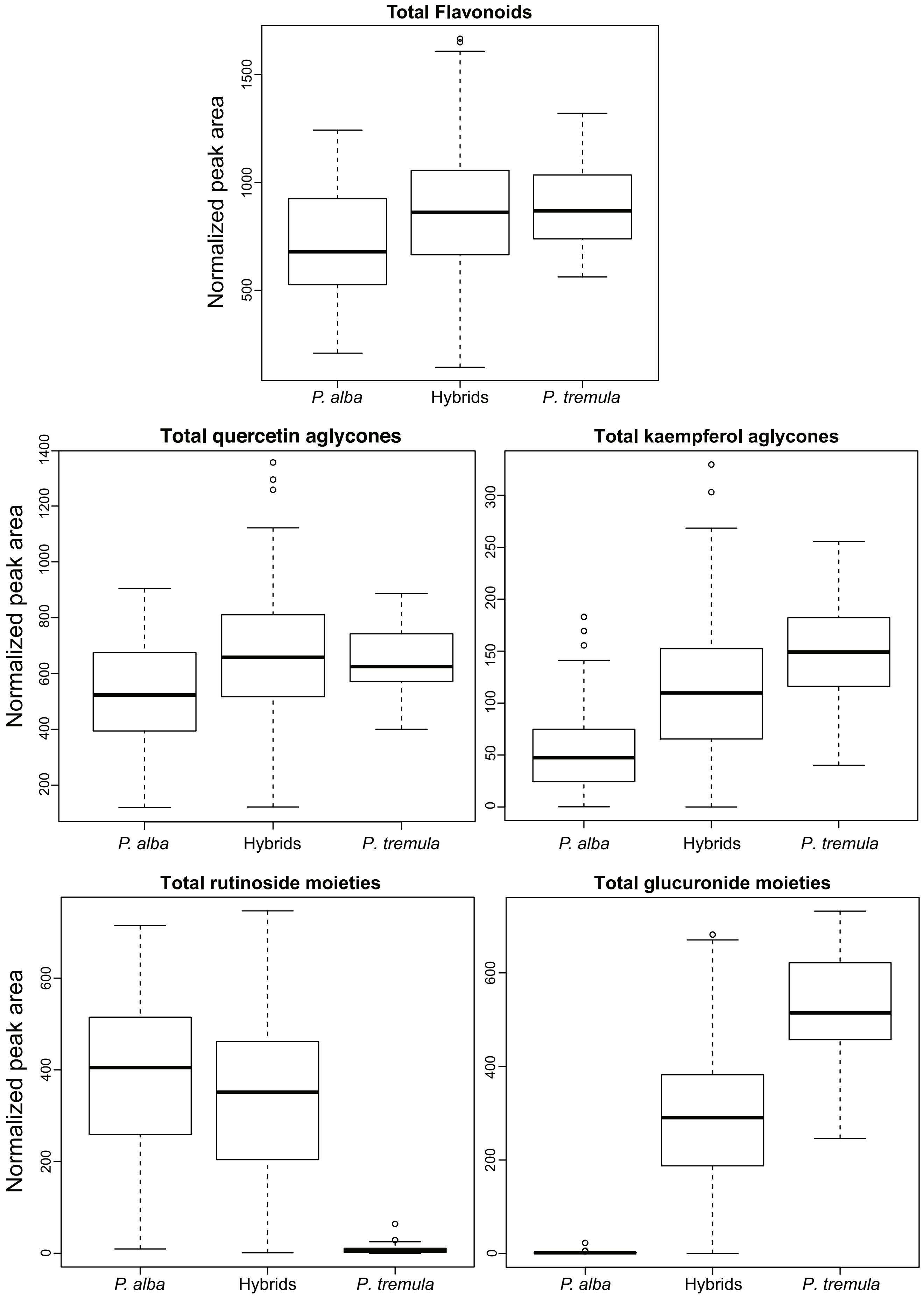

Supplement: S2 Fig — Normalized peak areas used as measures of relative abundances of compounds were summed within groups of aglycones and moieties for P. alba, P. tremula and their hybrids from three natural hybrid zones. For compound diversity see S3 Table. (PDF) [file pone.0128200.s002.pdf]
