# Supplementary material for: Effects of Hybridization and Evolutionary Constraints on Secondary Metabolites: The Genetic Architecture of Phenylpropanoids in European Populus Species
Source: PLoS One. 2015 May 26;10(5):e0128200. doi: 10.1371/journal.pone.0128200 (PMC4444209; doi:10.1371/journal.pone.0128200)
Supplement: S1 Materials — B) Molecular genetic analysis of common garden seedlings. (PDF) [file pone.0128200.s003.pdf]

S1 Materials

A) High-throughput quantification of phenylpropanoids in natural populations

Metabolic fingerprinting, the quantification of specific targeted phytochemical traits, was performed through Ultra-High-Pressure Liquid Chromatography coupled with Quadrupole-Time-Of-Flight Mass Spectrometry (UHPLC-QTOF-MS) on an Acquity UHPLC system (Waters corp., Milford, USA) coupled with an electrospray source Synapt G2 QTOF-MS (Waters corp.). The separation was achieved on a reversed-phase Acquity BEH C18 column (50x2.1 mm column, particle size 1.7 µm, Waters corp.). Solvents were A= water + 0.1% vol. formic acid; B = acetonitrile + 0.1% vol. formic acid. The gradient program at flow of 0.4 mL⁄min of solvents was: 2-40% B in 4 min, 40-100% B in 2 min, holding at 100% B for 1.5 min followed by re-equilibration at 2% B for 2 min. The column temperature was maintained constant at 30°C and the injection volume of leaf extract was 5 µl. Mass over charge (m/z) data from the QTOF-MS were obtained in negative ion modes ($[M-H]^{-}$, S3 Table) over an *m/z* range of 85-1200 Da with the following parameters: capillary voltage at -2.5 kV, cone voltage at 25 V, source temperature at 120°C, desolvation gas temperature at 330°C, desolvation gas flow 800 L⁄hour. Argon was used as collision gas at a flow of 2.1mL⁄min. Internal calibration of the instrument was obtained by infusing a solution of leucinenkephaline at 600 ηg⁄mL at a flow rate of 10 µL⁄min through the Lock Spray™ probe. The abundance of targeted secondary metabolites was quantified in a relative manner on the basis of peak areas obtained from the QTOF-MS normalized over the total spectrum area. For each compound, the corresponding most abundant ion (e.g. ion of the molecular species, formate adduct or dimer) was considered for quantification. (S3 Table). The identification of targeted compounds by High-Resolution tandem Mass Spectrometry (HR-MS/MS) and comparison to pure standards is documented in [1].

B) Molecular genetic analysis of common garden seedlings

Leaf samples from each common garden seedling were dried with silicagel. Total genomic DNA was extracted with the DNeasy Plant Mini Kit (Qiagen) and quantified with a NanoDrop photometer (Thermo scientific). Three multiplex reaction mixes with six marker loci each were designed by selecting highly informative microsatellites in natural hybrid populations of *Populus alba* and *P. tremula* (S2 Table). The multiplexed microsatellites were amplified in 15 µl Polymerase Chain Reactions (PCR) including 10ng of DNA with Type-it Microsatellite PCR kit (Qiagen). Concentrations of primers including fluorescent dyes (FAM, VIC, NED and PET from Applied Biosystems) were adjusted in the

primer mix to adjust for differing signal intensities among markers. PCR conditions were: denaturation for 5 min at 95°C followed by 28 cycles consisting of a denaturation step of 30 sec at 95 °C, an annealing step of 90 sec at 57 °C, and an extension step of 30 sec at 72 °C, followed by a final extension step of 30 min at 60 °C. Amplified fragments were analyzed on an Applied Biosystem 3130 Genetic Analyzer with LIZ (Applied Biosystem) as internal size standard. Two marker loci were dropped from downstream analyses because missing data frequencies were too high. For the remaining 16 markers, numbers of alleles (Table S2) were calculated with Microsatellite Analyzer [2] and gene diversity was estimated with the FSTAT software [3]. The genomic composition of the common garden seedlings was estimated through genomic admixture proportions (Q) based on 16 multiplexed microsatellite markers (Table S2) with the Bayesian approach implemented in the STRUCTURE software [4-6] using an admixture model and parental species samples (42 *P. alba*, 68 *P. tremula*) from the Ticino river hybrid zone as references. A burn-in of 50 000, followed by 100 000 iterations, a prior mean of F_k of 0.7 and k=2 populations were specified in STRUCTURE runs. Individual common garden plants (S1 Table) were characterized as *P. alba* (Q>0.95), *P. x canescens* hybrids (0.05<Q<0.95) and *P. tremula* (Q<0.05) according to their admixture proportions. The correlation of paternity (Cp) was calculated following [7] using the MLTR software to estimate percentages of full-siblings (=seedlings sharing the same father) in the open pollinated families. The microsatellite genotype data were also used to confirm the absence of seed contaminations (unrelated genotypes) within the families based on codominant inheritance of the markers, following Lexer et al. [8]. Inspection of the maternal genotypes (=seed parents of the open pollinated families) revealed two cases of exact genotype matches between spatially separated mother trees, raising the possibility that these mothers form part of ancient clones. Thus, all common garden analyses in this paper were carried out for 15 families (as expected from the seed harvest) and 13 families (progeny of matching mother pairs merged). As the results were highly similar, only results from the 15 family dataset are discussed throughout the paper. This was regarded more appropriate, since seeds from each maternal tree had been exposed to that tree's particular microenvironment during development, and the maternal genotype matches do not exclude the existence of genetic differences at other (untyped) loci or somaclonal mutations in the genomes of ancient clones.

References

1. Caseys C, Glauser G, Stolting KN, Christe C, Albrectsen BR, et al. (2012) Effects of interspecific recombination on functional traits in trees revealed by metabolomics and genotyping-by-resequencing. Plant Ecology & Diversity 5: 457-471.
2. Dieringer D, Schlötterer C (2003) Microsatellite analyser (MSA): a platform independent analysis tool for large microsatellite data sets. Molecular Ecology Notes 3: 167-169.
3. Goudet J (1995) FSTAT (Version 1.2): A computer program to calculate F-statistics. Journal of Heredity 86: 485-486.
4. Pritchard JK, Stephens M, Donnelly P (2000) Inference of population structure using multilocus genotype data. Genetics 155: 945-959.
5. Falush D, Stephens M, Pritchard JK (2003) Inference of population structure using multilocus genotype data: Linked loci and correlated allele frequencies. Genetics. pp. 1567-1587.
6. Falush D, Stephens M, Pritchard JK (2007) Inference of population structure using multilocus genotype data: dominant markers and null alleles. Molecular Ecology Notes 7: 574.
7. Ritland K (2002) Extensions of models for the estimation of mating systems using n independent loci. Heredity 88: 221-228.
8. Lexer C, Heinze B, Steinkellner H, Kampfer S, Ziegenhagen B, et al. (1999) Microsatellite analysis of maternal half-sib families of *Quercus robur*, pedunculate oak: detection of seed contaminations and inference of the seed parents from the offspring. Theoretical and Applied Genetics 99: 185-191.
9. Lindtke D, Buerkle CA, Barbará T, Heinze B, Castiglione S, et al. (2012) Recombinant hybrids retain heterozygosity at many loci: new insights into the genomics of reproductive isolation in *Populus*. Molecular Ecology 21: 5042-5058.
