# Supplementary material for: Effects of Hybridization and Evolutionary Constraints on Secondary Metabolites: The Genetic Architecture of Phenylpropanoids in European Populus Species
Source: PLoS One. 2015 May 26;10(5):e0128200. doi: 10.1371/journal.pone.0128200 (PMC4444209; doi:10.1371/journal.pone.0128200)
Supplement: S1 Table — Hybrid individuals and plants from each parental species (P. alba and P. tremula) were characterized with 77 microsatellite DNA markers in a previous study [23]. The common garden plants were genotyped with 16 microsatellites for the present study (see text and S2 Table for details). Assignment to each taxon was based on Bayesian genomic admixture proportions Q as described in main text. (PDF) [file pone.0128200.s004.pdf]

S1 Table. Number of individuals studied in three natural hybrid zones. Hybrid individuals and plants from each parental species (*P. alba* and *P. tremula*) were characterized with 77 microsatellite DNA markers in a previous study [9]. The common garden plants were genotyped with 16 microsatellites for the present study (see text and S2 Table for details). Assignment to each taxon was based on Bayesian genomic admixture proportions Q as described in main text.

	Hybrids	*P. alba*	*P. tremula*
Ticino river hybrid zone	109	15	15
Danube hybrid zone	20	21	16
Tisza river hybrid zone	34	15	15
Common garden trial	104	9	20
