# Supplementary material for: Effects of Hybridization and Evolutionary Constraints on Secondary Metabolites: The Genetic Architecture of Phenylpropanoids in European Populus Species
Source: PLoS One. 2015 May 26;10(5):e0128200. doi: 10.1371/journal.pone.0128200 (PMC4444209; doi:10.1371/journal.pone.0128200)
Supplement: S2 Table — Information for the 16 microsatellite marker loci used to identify parental species and hybrids and to estimate the correlation of paternity (Cp) in the common garden. These 16 microsatellites are a subset of the genome-wide marker panel used for admixture mapping in natural hybrid zones and are fully described [23]. Localization on chromosomes, allele frequency differential (delta) between the parental reference populations of the Italian hybrid zone [23], number of alleles (NA) and gene diversity (He) in the common garden trial are indicated. (PDF) [file pone.0128200.s005.pdf]

S2 Table. Molecular marker used to characterize common garden seedlings

Information for the 16 microsatellite marker loci used to identify parental species and hybrids and to estimate the correlation of paternity (Cp) in the common garden. These 16 microsatellites are a subset of the genome-wide marker panel used for admixture mapping in natural hybrid zones and are fully described in Lindtke et al. [9]. Localization on chromosomes, allele frequency differential (delta) between the parental reference populations of the Italian hybrid zone [9], number of alleles (N_A) and gene diversity (He) in the common garden trial are indicated.

Name	Chr.	Delta	N_A	He
G124	1	0.78	7	0.794
ASP376	1	0.94	6	0.721
G1158	2	1	6	0.584
G1255	5	1	4	0.546
0G1831	6	0.72	10	0.824
G1074	6	0.98	4	0.517
O26	6	0.71	7	0.700
O167	6	0.96	2	0.498
ASP933	6	1	7	0.747
O190	6	0.95	4	0.519
ASP322	6	1	15	0.857
G2020	10	0.77	17	0.867
G1574	10	0.98	6	0.634
G1812	14	0.85	7	0.787
G1894	15	0.97	7	0.704
O214	18	0.83	4	0.463
