# Supplementary material for: Effects of Hybridization and Evolutionary Constraints on Secondary Metabolites: The Genetic Architecture of Phenylpropanoids in European Populus Species
Source: PLoS One. 2015 May 26;10(5):e0128200. doi: 10.1371/journal.pone.0128200 (PMC4444209; doi:10.1371/journal.pone.0128200)
Supplement: S6 Table — Contingency tables presenting counts of the presence (yes) or absence (no) of an excess of interspecific heterozygote [23], for all 67 codominant genetic markers (S5 Table) studied in natural hybrid zones of P. alba and P. tremula, for markers representing putative QTL for salicinoids and flavonoids, and for all 34 markers representing putative phytochemical QTL in the present study. (PDF) [file pone.0128200.s009.pdf]

S6 Table. Excess of interspecific heterozygosity linked with phytochemical QTL.

Contingency tables presenting counts of the presence (yes) or absence (no) of an excess of interspecific heterozygotes [9], for all 67 codominant genetic markers (S5 Table) studied in natural hybrid zones of *P. alba* and *P. tremula*, for markers representing putative QTL for salicinoids and flavonoids, and for all 34 markers representing putative phytochemical QTL in the present study.

**Ticino hybrid zone**<br>Heterozygote excess	All markers	Salicinoids	Flavonoids	All 34 QTL
Yes	30	**8**	**11**	**21**
No	37	**8**	**10**	**22**

**≥ 2 hybrid zones**<br>Heterozygote excess	All markers	Salicinoids	Flavonoids	All 34 QTL
Yes	22	4	**9**	**15**
No	45	12	**12**	**28**

Note: Chlorogenic acids were not tested individually, because the number of detected QTL was low (S5 Table). Patterns were examined for the Italian hybrid zone (the population of origin of our common garden trial) and across hybrid zones. Bold type identifies comparisons with heterozygote excess. For these comparisons, Fisher's exact test could not reject the independence of count distributions for QTL-linked markers (salicinoids, flavonoids, all 34 QTL) *versus* all codominant markers, i.e. there was an elevated number of markers with heterozygosity excess among the phytochemical QTL.
